# Delaying chloroplast turnover increases water-deficit stress tolerance through the enhancement of nitrogen assimilation in rice

**DOI:** 10.1093/jxb/erx247

**Published:** 2017-07-27

**Authors:** Nir Sade, Kamolchanok Umnajkitikorn, Maria del Mar Rubio Wilhelmi, Matthew Wright, Songhu Wang, Eduardo Blumwald

**Affiliations:** 1 Department of Plant Sciences, University of California, Davis, CA, USA; 2 CAS Center for Excellence in Molecular Plant Sciences, Chengdu Institute of Biology, Chinese Academy of Sciences, Chengdu, China

**Keywords:** CHLOROPLAST VESICULATION, nitrogen assimilation, photosynthesis, photorespiration, stress-induced senescence, water stress

## Abstract

Abiotic stress-induced senescence in crops is a process particularly affecting the photosynthetic apparatus, decreasing photosynthetic activity and inducing chloroplast degradation. A pathway for stress-induced chloroplast degradation that involves the *CHLOROPLAST VESICULATION* (*CV*) gene was characterized in rice (*Oryza sativa*) plants. *OsCV* expression was up-regulated with the age of the plants and when plants were exposed to water-deficit conditions. The down-regulation of *OsCV* expression contributed to the maintenance of the chloroplast integrity under stress. *OsCV*-silenced plants displayed enhanced source fitness (i.e. carbon and nitrogen assimilation) and photorespiration, leading to water-deficit stress tolerance. Co-immunoprecipitation, intracellular co-localization, and bimolecular fluorescence demonstrated the *in vivo* interaction between OsCV and chloroplastic glutamine synthetase (OsGS2), affecting source–sink relationships of the plants under stress. Our results would indicate that the OsCV-mediated chloroplast degradation pathway is involved in the regulation of nitrogen assimilation during stress-induced plant senescence.

## Teaser

Silencing of the CHLOROPLAST VESICULATION gene leads to plant stress tolerance and increased nitrogen assimilation in rice.

## Introduction

During senescence, plants undergo a process of tissue degeneration and nutrient recycling to support the growth of new plant organs and reproduction (Quirino *et al.*, 2000; Hortensteiner and Feller, 2002). Autophagy and senescence-associated vacuoles (SAVs) play critical roles in leaf age-dependent senescence (Xie *et al.*, 2015). Environmental stresses such as high salinity and water deficit, among others, promote early plant senescence (Diaz-Mendoza *et al.*, 2016) limiting plant productivity and, as a consequence, reducing agricultural output (Zhu, 2016). This process, known as stress-induced senescence, affects particularly the photosynthetic apparatus, decreasing photosynthetic activity by promoting chloroplast degradation (Quirino *et al.*, 2000). There is a significant amount of research on the molecular, biochemical, and physiological signals that regulate leaf senescence. Hormone-dependent signaling as well as WRKY and NAC transcription factors (TFs) have been reported to regulate the senescence process (Balazadeh *et al.*, 2010; Thomas and Ougham, 2014; Raineri *et al.*, 2015; Rinerson *et al.*, 2015). Cytokinins (CKs) and abscisic acid (ABA) were suggested to be negative and positive regulators of stress-induced senescence, respectively (Peleg and Blumwald, 2011).

It has been shown that delaying leaf senescence may result in extended photosynthetic capacity and therefore result in changes in plant source–sink relationships, usually associated with grain yield changes during stress (Gregersen *et al.*, 2013). A number of strategies for the delay of stress-induced senescence resulted in enhanced plant stress tolerance. Peleg *et al.* (2011) showed that stress-induced CK synthesis in rice increased tolerance to water deficit. CK synthesis modified source–sink relationships in rice, resulting in improved source strength and leading to higher grain yields under stress. Another strategy for delayed senescence, referred to as a stay-green (SGR) phenotype (Thomas and Ougham, 2014), is mainly associated with alterations of chlorophyll metabolism. Delayed chlorophyll degradation and/or chlorophyll overproduction (Hortensteiner and Krautler, 2011) can play important role(s) in stress tolerance (Gregersen *et al.*, 2013; Jagadish *et al.*, 2015). In addition, senescence-associated TFs have been shown to be associated with stress-induced senescence and stress tolerance. For example, ANAC092 and ONAC106, members of the NAC family of TFs, are involved in salt-induced chloroplast degradation and early senescence in Arabidopsis and rice plants, respectively (Balazadeh *et al.*, 2010; Sakuraba *et al.*, 2015). The above-mentioned reports and others support the notion that delaying leaf senescence can contribute to plant stress tolerance. Nevertheless, both positive and negative effects of delayed senescence on yield quantity and quality (depending on the developmental stage of the crop at the time of the stress episode) have been reported (Gregersen *et al.*, 2013; Jagadish *et al.*, 2015).

Recently, a protein named CHLOROPLAST VESICULATION (CV) was suggested to regulate stress-induced senescence-associated chloroplast degradation in Arabidopsis (Wang and Blumwald, 2014). *AtCV* is a nuclear gene encoding a protein that interacts with thylakoid membrane-bound proteins (i.e. PsbO1 in PSII, CYP20 in PSI, etc.). It was shown that CV overexpression led to chloroplast degradation by destabilization of the photosynthetic apparatus during stress. Upon stress, AtCV-containing vesicles (CCVs) mobilize thylakoid and stromal proteins to the vacuole for degradation through a pathway that is independent of authophagy and senescence-associated vacuoles (SAVs) (Wang and Blumwald, 2014). In agreement with this notion, *CV* silencing in Arabidopsis resulted in chloroplast stability during stress and enhanced stress tolerance (Wang and Blumwald, 2014). Although the cellular pathways regulating *AtCV* chloroplast degradation have been described, the role(s) of CV in the regulation of carbon (C):nitrogen (N) metabolism under stress is not yet clear. Chloroplasts are the main source for C and N assimilation, and >70% of the leaf N is located at the chloroplasts, thus making the chloroplast an important plant nutrient storage unit (Hortensteiner and Feller, 2002). C assimilation at the chloroplast is highly co-ordinated with N assimilation (Nunes-Nesi *et al.*, 2010). Thus, key enzymes associated with N assimilation and remobilization such as cytosolic nitrate reductase (NR), chloroplastic glutamine synthetase (GS2), and mitochondrial glutamate dehydrogenase (GDH) are affected by senescence, stress, and chloroplast degradation (Masclaux-Daubresse *et al.*, 2006, 2010), suggesting that chloroplast stability may play a major role in controlling certain aspects of N assimilation and remobilization. Upon senescence and concurrent with chloroplast degradation, a marked decrease in N uptake and assimilation was observed, following an enhancement in N remobilization from senescent source leaves to sinks (Havé *et al.*, 2017). In agricultural crops growing under adverse environmental conditions, stress-induced senescence triggers the premature remobilization of nutrients from sources to sinks, leading to significant yield losses. Several reports supported the notion of a crosstalk between N assimilation, photosynthesis, and stress tolerance. Thus, under water-deficit stress, a strong correlation between the expression/activity of cytoplasmic NR and photosynthesis was observed (Foyer *et al.*, 1998). The delay of source senescence in rice, through the overexpression of CK synthesis, increased N assimilation and led to increased grain yield under water deficit (Reguera *et al.*, 2013). In addition, the overexpression of chloroplastic GS, a key enzyme in ammonia assimilation, resulted in enhanced salt tolerance in rice (Hoshida *et al.*, 2000). Interestingly, higher levels of GS were detected in Arabidopsis *cv* knockdowns under water-deficit conditions (Wang and Blumwald, 2014).

Although CV proteins were found in most plants species (Wang and Blumwald, 2014), their role(s) in N assimilation, source–sink relationships, and yield production in crop plants is not clear. We hypothesized that given that under abiotic stress, CV proteins play a significant role in chloroplast degradation and premature leaf senescence, *OsCV* silencing would enhance plant water stress tolerance by maintaining chloroplast function and C and N assimilation during the stress episode. Our results reveal the role of CV in the turnover of enzymes that are associated with N assimilation during stress, leading to decreased N assimilation and affecting photorespiration. Also, our results support the notion that delaying stress-induced senescence by maintaining chloroplast stability is a viable strategy for enhancing grain yield under stress conditions.

## Materials and methods

### Plant material and growth conditions

Seeds of wild-type rice (*Oryza sativa* japonica ‘Kitaake’) and transgenic plants were germinated on moist germination paper for 10 d at 28 °C in the dark. Seedlings were then transplanted into 2 liter pots filled with equal parts of coarse perlite and coarse vermiculite. Greenhouse conditions were kept at 12 h/2 h day/night and 28 °C/20 °C. Plants were fertilized daily with a solution (N 75 ppm, P 20 ppm, K 75 ppm, Ca 27 ppm, Mg 17 ppm, S 65 ppm, Fe 1.50 ppm, Mn 0.50 ppm, Zn 0.05 ppm, Mo 0.01 ppm, Cu 0.02 ppm) until the beginning of the flowering stage (panicle exsertion). Water-deficit stress treatments were applied by withholding water (starting at the tillering stage) for ~10 d until visual stress symptoms (i.e. leaf rolling) appeared (10–15% relative soil water content); plants were then re-watered for 2 d and water was withheld again as described above. The water stress/re-water cycle was repeated three times (water stress cycles) before plants were re-watered and grown under normal conditions until harvest. This strategy was based on the alternate wetting and drying (AWD) watering regimes used in rice fields (Peng, 2011). The youngest fully expanded leaves at the end of the first dry cycle were sampled for transcriptomics and metabolomics, and measurements of gas exchange and enzymology were taken at this point. The youngest fully expanded leaves for measurements of chlorophyll content, protein content, and samples for electron microscopy were taken at the end of the second cycle of the stress, while samples for malondialdehyde (MDA) measurements were taken at the end of the third cycle of the stress period. For the estrogen-induced experiments, transgenic plants were sprayed daily for 10 d with either DMSO or 50 µM β-estradiol (E8875 Sigma Aldrich) in DMSO.

### Gas-exchange measurements

Photosynthesis measurements were recorded in plants inside a controlled-environment chamber using a Li-6400 portable gas-exchange system (LI-COR). Photosynthesis was induced by saturating light (1200 μmol m^−2^ s^−1^) with 400 μmol mol^−1^ CO_2_ surrounding the leaf (*C*_a_). The amount of blue light was set to 10% photosynthetically active photon flux density to optimize stomatal aperture. Temperature was set to 28 °C.

### Metabolite profiling

Metabolites were analyzed in the youngest fully expanded leaves of wild-type and transgenic plants grown under well-watered and water-deficit stress conditions. Samples were submitted to the West Coast Metabolomics Center at UC Davis and extracted, measured, and analyzed by GC-MS (Gerstel CIS4 with a dual MPS Injector/Agilent 6890 GC-Pegasus III TOF MS) as described before (Weckwerth *et al.*, 2004). Processes for the integrated extraction, identification, and quantification of metabolites were performed according to Fiehn *et al.* (2008). There were no significant differences in the examined metabolites under well-watered conditions (data not shown). Therefore, for simplicity, results are presented for water-deficit stress conditions.

### Enzyme assays

Enzyme activities were determined in the youngest fully expanded leaves of wild-type and transgenic plants subjected to water-deficit stress and collected in the morning (09.00–10.00 h). Whole leaves from each plant were ground with liquid nitrogen and the powder was aliquoted. Enzyme activities are expressed as moles of metabolite generated/consumed per milligram of protein per unit of time. For NR activity, frozen leaf powder was homogenized in the presence of 1 ml of buffer containing 50 mM KH_2_PO_4_-KOH buffer pH 7.5, 2 mM EDTA, 2 mM DTT, and 1% polyvinylpolypyrrolidone. Extracts were centrifuged at 20 000 *g* for 20 min at 4 °C. NR activity was measured according to Kaiser and Lewis (1984) with some modifications. The reaction was initiated by the addition of 700 µl of reaction buffer (50 mM KH_2_PO_4_-KOH buffer, pH 7.5, 10 mM KNO_3_, and 0.1 mM NADH) to 100 µl of total soluble proteins. Samples were incubated at 28 °C for 15 min, and controls were boiled before incubation with the reaction buffer. The reactions were stopped, and NO_2_^−^ was determined. Deamination activity of GDH was assessed according to protocols described previously (Loyola Vargas and Dejimenez, 1984). GS was determined according to O’Neal and Joy (1973).

### Malondialdehyde measurements

The youngest fully expanded leaves were homogenized with 5 ml of 50 mM NaH_2_PO_4_-Na_2_HPO_4_ buffer pH 7.5 and centrifuged at 20 000 *g* for 25 min. For measurements of MDA concentration, 4 ml of 20% trichloroacetic acid containing 0.5% thiobarbituric acid were added to a 1 ml aliquot of the supernatant. The mixture was heated at 95 °C for 30 min, quickly cooled in ice, and then centrifuged at 10 000 *g* for 10 min. The absorbance of the supernatant was measured at 532 nm and 600 nm. The results are shown as ΔOD_532_ mg^–1^ DW.

### Chlorophyll measurements

For chlorophyll measurements, the youngest fully expanded leaves were weighed and ground in liquid N_2_. Chlorophyll was extracted in 80% acetone, and the absorbance at 663 nm and 645 nm was measured (Synergy™ Mx Microplate Reader; BioTek, USA). Total chlorophyll contents were calculated as described elsewhere (Porra, 2002).

### Protein quantification

The Bradford assay (Bradford, 1976) was used for protein quantification using BSA as a standard.

### Quantitative PCR analysis

RNA was extracted from leaves of wild-type and transgenic rice plants under well-watered and water-deficit stress conditions. First-strand cDNA synthesis, primer design, and quantitative PCR were performed as described before (Peleg *et al.*, 2011). The different sets of primers used for the amplification of the target genes are listed in Supplementary Table S1 at *JXB* online. Analysis of the relative gene expression was performed according to the comparative cycle threshold (2^−ΔΔCT^) method (Livak and Schmittgen, 2001) and calibrated using transcript values relative to the endogenous rice transcription elongation factor (TEF) gene (Peleg *et al.*, 2011; Reguera *et al.*, 2013; Tamaki *et al.*, 2015).

### RNA sequencing

RNA sequencing and analysis were performed by the DNA Technologies Core and Bioinformatics Core at the University of Davis, California, USA. Briefly, RNASeq libraries were prepared using the Illumina (bio scientific NEXTflex Rapid Directional qRNA-Seq Kit-Set A) mRNA library kit using the standard protocol. These libraries were then sequenced for a single read (SR90) on an Illumina HiSeq 4000 system (HiSEQ Control Software 2.2.38, RTA 1.18.61) following the standard rapid sequencing workflow. Raw reads were pre-processed using the expHTS pipeline https://github.com/msettles/expHTS); briefly: custom versions of FLASH2 https://github.com/dstreett/FLASH2) and Sickle (https://github.com/dstreett/sickle) for adaptor and quality trimming, respectively, with a Python wrapper to perform duplicate marking and track statistics between each step. Then, STAR version 2.5.1b (Dobin *et al.*, 2013) was used to align pre-processed reads to the *O. sativa* IRGSP-1.0 reference genome. Then, strand-specific per-gene read counts were extracted from STAR’s ReadsPerGene.out.tab files using BASH commands, in order to obtain the count table for statistical analysis. Prior to analysis, genes with <0.5 counts per million reads in all samples were filtered. Differential expression analyses were conducted using the Limma-Voom Bioconductor pipeline [limma version 3.28.21 (Ritchie *et al.*, 2015), edgeR version 3.14.0 (Robinson *et al.*, 2010), in R 3.3.1: R Core Team], using a two-factor model factored for genotype, treatment, and their interaction. Gene Ontology (GO) enrichment analyses were conducted using Kolmogorov–Smirnov tests as implemented in the Bioconductor package topGO, version 2.24.0 (Alexa and Rahnenfuhrer, 2009). Kyoto Encyclopedia of Genes and Genomes (KEGG) enrichment analyses were conducted using Wilcoxon rank sum tests and the Bioconductor package KEGGREST, version 1.12.3 (Tenenbaum, 2016).

There were no significant differences in the expression of the examined genes under well-watered conditions (data not shown). Therefore, for simplicity, results are presented for water-deficit stress conditions.

### Electron microscopy

For standard TEM, the youngest fully expanded leaves were fixed in Karnovsky’s fixative [2.0% paraformaldehyde and 2.5% glutaraldehyde (Electron Microscopy Sciences, Hatfield, PA, USA) in 0.1 M sodium phosphate buffer, pH 7.4]. Samples were post-fixed with 1% OsO_4_ in the same buffer. Leaves were dehydrated in increasing concentrations of ethyl alcohol ending with two changes of propylene oxide, then infiltrated with an Epon/Aradite mixture resin. Ultrathin cross-sections (70 nm) of the rice mesophyll cells were obtained using a Leica Ultracut UCT ultramicrotome and stained with uranyl acetate followed by lead citrate. The samples were observed with a Philips CM120 Biotwin lens (F.E.I.).

### Immunoblot analyses

The youngest fully expanded leaf tissues were weighed, frozen in liquid N_2_, and ground in 3 vols of extraction buffer (50 mM HEPES, 100 mM NaCl, 10 mM KCl, and 0.4 M sucrose) containing 1 mM phenylmethylsulfonyl fluoride (PMSF) and protease inhibitor. Total proteins were denatured by mixing with Laemmli sample buffer and then separated by SDS–PAGE, transferred to a polyvinylidene difluoride membrane (Bio-Rad), and probed as described previously (Wang *et al.*, 2011). Antibodies raised against green fluorescent protein (GFP; NB600-308) were obtained from Novus Biologicals (Littleton, CO, USA). Horseradish peroxidase-conjugated secondary antibodies were purchased from Santa Cruz Biotechnology (Dallas, TX, USA).

### Fluorescence and confocal microscopy

Fluorescence microscopy was performed using an inverted Zeiss LSM 710 confocal laser scanning microscope (Carl Zeiss, Oberkochen, Germany) equipped with a ×40 water immersion objective. The excitation wavelength/emission were as follows for GFP (488 nm/500–530 nm), yellow fluorescent protein (YFP; 514 nm/527–572 nm), cyan fluorescent protein (CFP; 405 nm/463–498 nm), red fluorescent protein (RFP; 561 nm/600–660 nm), and chlorophyll (633 nm/650–720 nm).

### Immunoprecipitation and LC-MS/MS

One-week-old seedlings of the wild type and transgenic plants *EST::OsCV-GFP* and *35s::CT-sGFP* (Izumi *et al.*, 2015) were cultured in MS/2 medium containing 20 µM β-estradiol or DMSO for 24 h. The shoots of the plants were harvested, ground in liquid N_2_, and incubated at 4 °C for 4 h with lysis buffer provided in the μMACS GFP Isolation Kit (Miltenyl Biotec, Bergisch Gladbach, Germany), containing protease inhibitor cocktail (Sigma-Aldrich, St. Louis, MO, USA) and 1 mM PMSF. Co-immunoprecipitation was performed using anti-GFP magnetic beads from the μMACS GFP Isolation Kit (Miltenyl Biotec) and incubating the cell lysate with beads at 4 °C for 4 h. LC-MS/MS analysis was performed at the Proteomic core facility of the University of California-Davis, as described previously (Shipman-Roston *et al.*, 2010). Scaffold (version Scaffold 4; www.proteomesoftware.com) was used to validate tandem MS-based peptide and protein identification. The results were filtered with a false discovery rate (FDR) of <0.5% on the peptide level and 1% on the protein level, with a minimum of two unique peptides required for identification (Fan *et al.*, 2016).

### Statistical analysis

The JMP (version 8.0) statistical package (SAS Institute) was used for statistical analyses. The experiments were based on a randomized complete block design.

### Constructs and generation of transgenic plants

All the constructs in this study were generated using the Gateway system (Invitrogen, Carlsbad, CA, USA).

For RNAi*CV* plants, the ORF of *OsCV* was amplified and cloned into pDONR207 by BP reaction. pDONR207-OsCV was recombined via LR reaction into pBHb7GW-I-WG-UBIL (https://gateway.psb.ugent.be/search) in both sense and antisense orientation, resulting in pBHb7-RNAiOsCV (RNAi*CV*).

For *EST:OsCV* plants, the ORF of *OsCV* excluding the stop codon (OS05G0575000) was amplified and fused with the GFP gene at the 3' terminus of *OsCV*. The fusion fragment (*OsCV–GFP*) was cloned into pDONR207 by BP reaction. pDONR207-OsCV-GFP was recombined via LR reaction into an estrogen-inducible system, pMDC7 (Curtis and Grossniklaus, 2003) resulting in pMDC7-OsCV-GFP.

The vectors were transformed into rice plants at the UC Davis plant transformation facility using standard transformation protocols. All transgenic plants were verified for the presence of the transgene using PCR (data not shown).

For *in vivo* OsCV and OsGS2 co-localization assay, the ORF of *OsCV* (OS05G0575000) excluding the stop codon was amplified from mature leaf cDNA and cloned into pDONR207 by BP reaction. pDONR207-CV was recombined via LR reaction into the destination vector pEarleyGate 102 (Earley *et al.*, 2006) for CFP fusion. Using the same strategy, the *OsGS2* gene (OS04G0659100) was fused with YFP of pEarleyGate 101.

For bimolecular fluorescence complementation (BiFC), the vectors pDEST-GWVYNE and pDEST-GWSCYCE from the Gateway-based BiFC vector systems (Gehl *et al.*, 2009) were employed to fuse OsCV (OS05G0575000) and OsGS2 (OS04G0659100) with the N-terminus of yellow fluorescent protein Venus (Venus^N^) or the C-terminus of super CFP (SCFP^C^) to obtain the constructs OsGS2–SCFP^C^ and OsCV–Venus^N^. All the constructs were introduced into *Agrobacterium tumefaciens* GV3101. Transient expression was performed in leaves of *Nicotiana benthamiana* as described previously (Li, 2011).

## Results

### *OsCV* is up-regulated upon senescence and stress-induced senescence

**Fig. 1.**
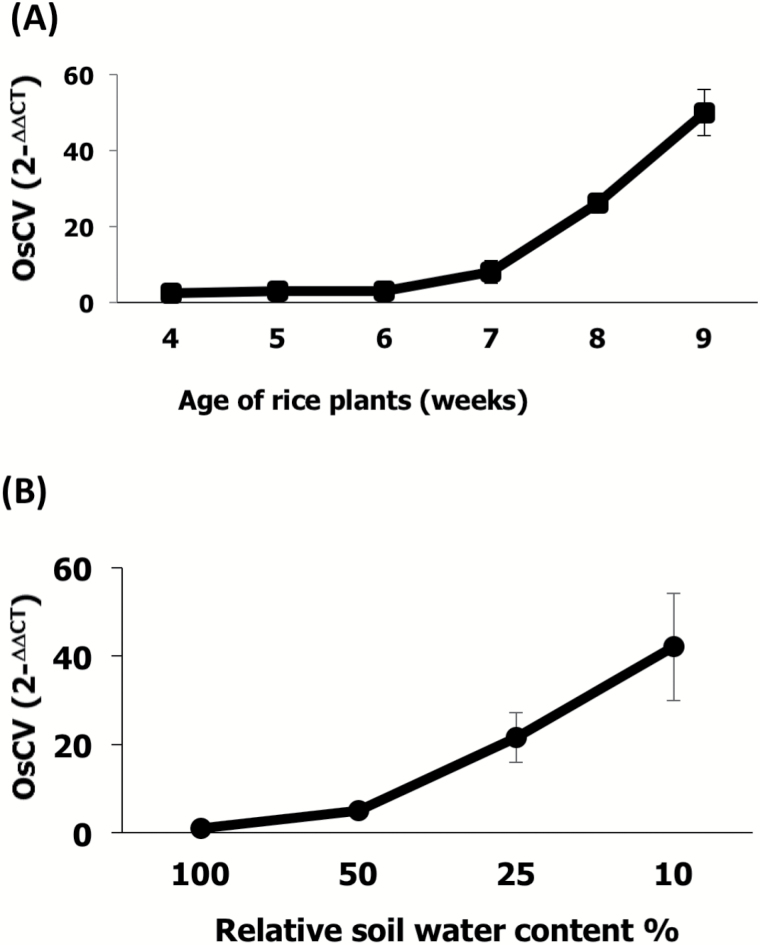
OsCV expression is induced by senescence and water stress. Quantitative RT-PCR analysis of *OsCV* gene expression in the youngest fully expanded leaves of wild-type plants in response to natural senescence (A) and water-deficit stress (B). Plant growth, water stress conditions, and quantitative RT-PCR conditions were as described in the Materials and methods. Values are the mean±SE (*n*=4 biological repetitions).

To assess the expression of *OsCV* across developmental stages, we measured *OsCV* transcript levels in wild-type plants growing under normal conditions from 4 weeks old (tillering stage) until 9 weeks old (grain-filling stage) (Fig. 1A). We also determined *OsCV* expression in the youngest fully expanded leaves of plants growing under decreasing soil water content to assess the effect of water deficit (Fig. 1B). *OsCV* expression was up-regulated with the age of the wild-type plants, reaching the highest expression at week 9 (Fig. 1A). Wild-type plants were exposed to different water stress conditions, by halting irrigation until the desired relative soil water content was attained. *OsCV* expression increased with the severity of the water-deficit stress condition, reaching a maximum at the lowest soil water content (10%) (Fig. 1B).

### Down-regulation of *OsCV* led to water stress tolerance

**Fig. 2.**
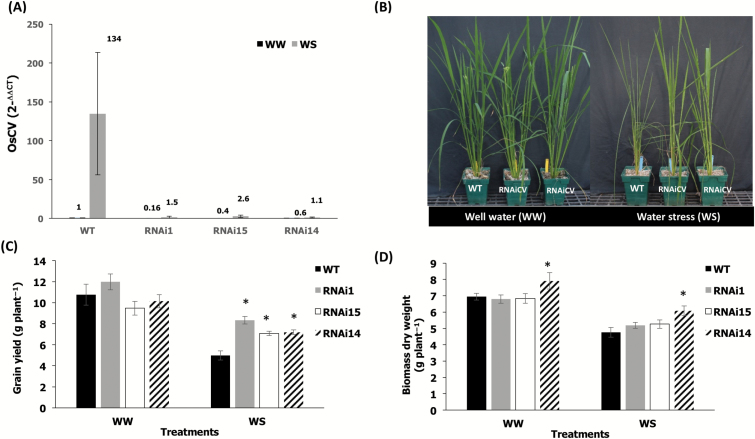
Effects of water stress on growth and yield. (A) Wild-type (WT) and transgenic RNAi*OsCV* silenced plants grown under well-watered conditions and subjected to water-deficit stress as described in the Materials and methods.(B) Quantitative RT-PCR analysis of *OsCV* expression in the youngest fully expanded leaves from WT plants and three independent RNAi*OsCV* silenced lines (RNAi1, RNAi15, and RNAi14) under well-watered (WW) and water stress (WS) conditions. (C) Grain yield. (D) Total biomass of plants harvested at the end of the experiment. Values are the mean±SE (*n*=14 biological repetitions). The data were analyzed using Student’s *t*-test. Asterisks indicate significant differences from the WT for each treatment (*P*≤0.05).

**Fig. 3.**
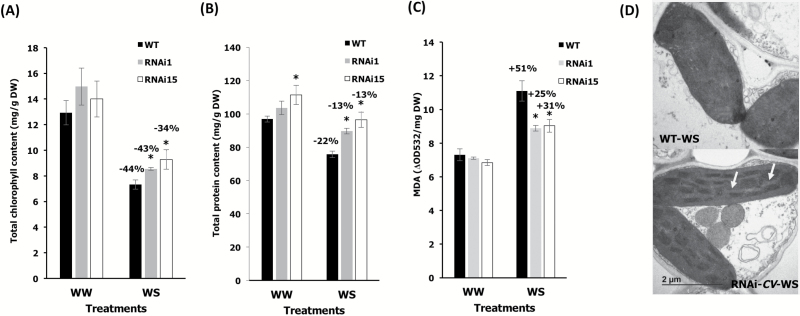
Effects of water stress on leaf stress parameters. (A) Total chlorophyll content, (B) total protein content, and (C) malondialdehyde (MDA) content of wild-type (WT) plants and silenced RNAi*OsCV* plants (RNAi1 and RNAi15). (D) Electron micrographs of chloroplasts from WT plants and the RNAi*OsCV* silenced plant line RNAi1 (RNAi*OsCV*) under water-deficit stress. Scale bars=2 μm. The arrows show well-organized grana thylakoids in RNAi*OsCV*. Values are the mean±SE (*n*=3–7 biological repetitions). The data were analyzed using Student’s *t*-test. Asterisks indicate significant differences from the WT for each treatment (*P*≤0.05). Percentages represent differences between control and stress conditions for each genotype.

In order to evaluate the roles of OsCV in rice, we generated *OsCV*-silenced transgenic rice using RNAi (Fig. 2A). *CV*-silenced plants displayed enhanced vigor under water-deficit stress (Fig. 2B) and an improved yield performance under stress (Fig. 2C). The above-ground biomass was slightly higher in *CV*-silenced plants but not significantly different between all genotypes under the tested conditions (Fig. 2D). We then assessed the response of both wild-type and RNAi*CV* rice plants to water-deficit stress (Fig. 3). Chlorophyll (Fig. 3A) and protein contents (Fig. 3B) of the youngest fully expanded leaves from RNAi*CV* were higher than for wild-type plants. We also analyzed MDA contents and showed that wild-type plants displayed higher lipid peroxidation than RNAi*CV* plants (Fig. 3C). Finally, we compared the chloroplast ultrastructure of wild-type and RNAi*CV* plants grown under control conditions and under water-deficit stress. Our results indicated the chloroplasts from wild-type plants exposed to a water-deficit stress lost the grana thylakoid organization while the chloroplasts from the CV-silenced plants remained intact (Fig. 3D).

### *OsCV* regulated photosynthesis and N metabolism

**Fig. 4.**
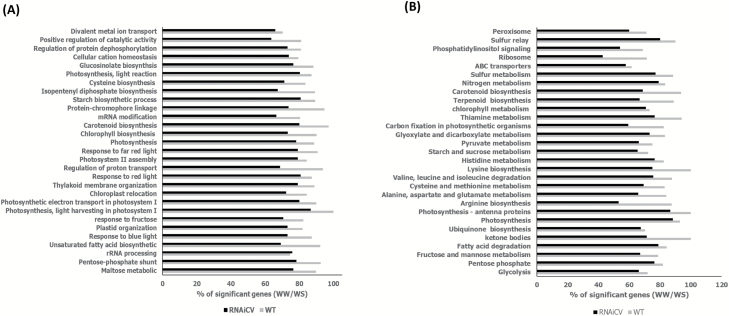
Gene Ontology enrichment of the most significant differentially expressed genes pathways in response to water-deficit stress (well-watered/water stress, WW/WS) for wild-type (WT) and RNAi*OsCV* plants (A) GO gene ontology (B) KEGG gene ontology.

**Fig. 5.**
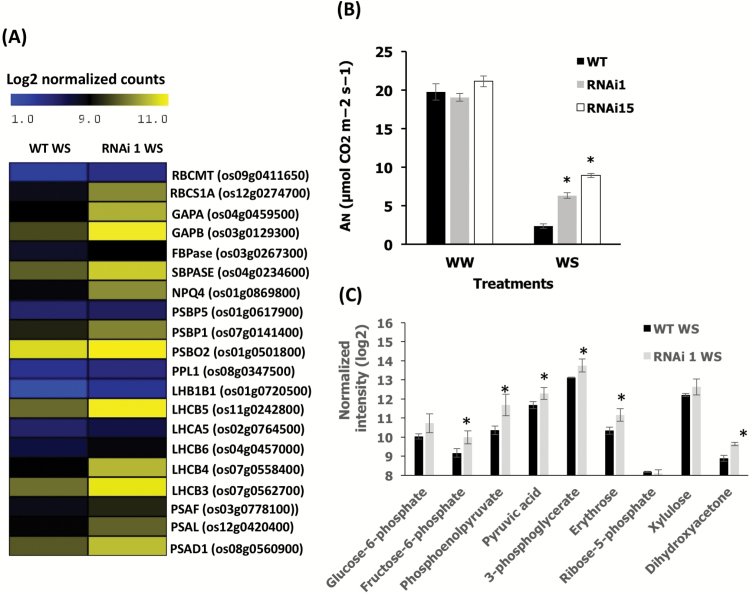
Effects of down-regulation of *OsCV* on photosynthesis. (A) A heatmap representation of selected photosynthesis-related genes (for full genes names, *P*-values, and expression values, see Supplementary Table S2) in wild-type (WT) and RNAi*OsCV* plants under water-deficit stress. (B) Analysis of carbon assimilation in flag leaves of WT and transgenic RNAi*OsCV* plants (lines RNAi1 and RNAi15). (C) Photosynthesis-associated metabolite contents from leaf tissue from WT and RNAi*OsCV* plants under water stress. Values are the mean±SE (*n*=3–8 biological repetitions). The data were analyzed using Student’s *t*-test. Asterisks indicate significant differences from the WT for each treatment (*P*≤0.05 except for F6pho *P*<0.06, pyruvic acid *P*<0.09, and 3PGA *P*<0.1).

To identify processes associated with the expression of *OsCV*, we performed gene expression profiling of wild-type and *OsCV*-silenced plants grown under control and water-deficit stress conditions. We examined the GO enrichment of genes showing a differential expression response to water stress (Fig. 4A) and we also mapped the differentially expressed genes into the KEGG database (Fig. 4B). Overall, the GO and KEGG analyses indicated that transcripts associated with photosynthesis, N and amino acid metabolism, and chloroplast structure and organization were differentially expressed between wild-type and RNAi*OsCV* plants in response to water-deficit stress. A detailed examination of photosynthesis-associated parameters revealed changes in wild-type plants as compared with RNAi*OsCV* plants under water stress (Fig. 5). In RNAi*OsCV* plants, representative genes from both ‘dark’ and ‘light’ reactions displayed higher levels of expression than in wild-type plants (Fig. 5A). These results were consistent with the higher photosynthetic activity, measured as the rates of CO_2_ assimilation, of the RNAi*OsCV* plants during water stress (Fig. 5B) and the increased leaf contents of primary metabolites associated with photosynthesis (Fig. 5C).

**Fig. 6.**
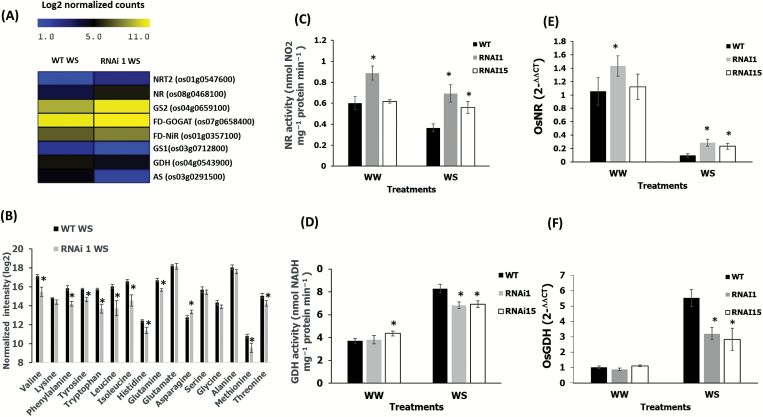
Down-regulation of *OsCV* resulted in alterations of N metabolism. (A) A heatmap representation of selected N metabolism-related genes (except AS *P*<0.053 and FD-NiR *P*<0.1; for full genes names, *P*-values, and expression values, see Supplementary Table S3) in wild-type (WT) and RNAi*OsCV* plants under water stress. (B) Analysis of free amino acids of WT and RNAi*OsCV* plants under water stress. (C) Activity and (E) expression of nitrate reductase in WT and RNAi*OsCV* plants (lines RNAi1 and RNAi15). (D) Deamination activity and (F) expression of glutamate dehydrogenase in WT and RNAi*OsCV* plants under water stress (lines RNAi1 and RNAi15). Values are the mean±SE (*n*=3–4 biological repetitions). The data were analyzed using Student’s *t*-test. Asterisks indicate significant differences from the WT for each treatment (*P*≤0.05).

**Fig. 7.**
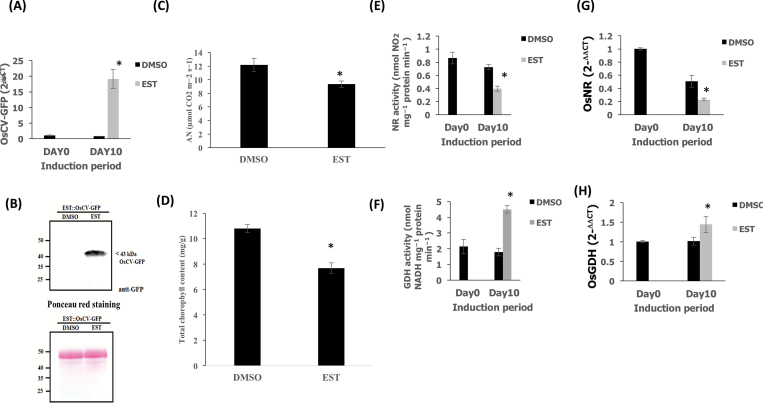
Overexpression of *OsCV* alters N metabolism. (A) Quantitative RT-PCR analysis of *OsCV* gene expression in the DMSO- and estrogen-treated plants transformed with *EST::OsCV-GFP*. (B) Immunoblot analysis of estrogen-induced OsCV–GFP expression using an anti-GFP antibody in DMSO (solvent control)- and estrogen-treated *EST::OsCV-GFP* plants. Ponceau S staining indicated the equal loading of total protein extractions for immunoblot analysis. (C) Analysis of carbon assimilation. (D) Total chlorophyll content (E), activity, and (G) expression of nitrate reductase in DMSO- and estrogen-treated *EST::OsCV-GFP* plants. (F) Activity (deamination) and (H) expression of glutamate dehydrogenase in DMSO- and estrogen-treated *EST::OsCV-GFP*. Values are the mean±SE (*n*=3–4 biological repetition). The data were analyzed using Student’s *t*-test. Asterisks indicate significant differences from DMSO-treated plant for each treatment (*P*≤0.05).

C and N assimilation are highly co-ordinated processes taking place mainly in chloroplasts, affecting source fitness and source–sink interactions (Reguera *et al.*, 2013). We examined the N assimilation process in both wild-type and RNAi*OsCV* plants under water-deficit stress (Fig. 6). The expression levels of genes encoding proteins associated with primary N assimilation were significantly higher in RNAi*OsCV* than in wild-type plants (Fig. 6A; Supplementary Fig. S1). This was the case for both cytosolic (e.g. NR) and chloroplastic proteins [e.g ferredoxin-glutamine oxoglutarate aminotransferase (Fd-GOGAT), GS2, and nitrite reductase (NiR)]. In contrast, genes associated with processes shown to direct amino acid synthesis [*GDH*, *GS1*, and *ASPARAGINE SYNTHETASE* (*AS*)], in conditions where N assimilation is decreased, were significantly higher in wild-type than in RNAi*OsCV* plants (Fig. 6A; Supplementary Fig. S1). Thus, RNAi*OsCV* plants were able to maintain primary N assimilation processes represented by higher *NR* and lower *GDH* expression and NR and GDH activities (Fig. 6C–F). Moreover, the water stress-induced inhibition of photosynthesis (Fig. 5) and the reduction in N assimilation (Fig. 6C, E) in the wild-type plants were accompanied by a dramatic accumulation of amino acids (Fig. 6B), suggesting a limitation in source fitness. To assess the effects of *OsCV* overexpression, transgenic plants overexpressing *OsCV* under the control of the β-estradiol-induced promoter (EST) were generated (*EST::CV-GFP*). Upon treatment with β-estradiol, *OsCV* expression was induced in the *EST::CV-GFP* transgenic plants (Fig. 7A). Western blots analysis of the transgenic plants treated with and without β-estradiol revealed the presence of the OsCV protein in the plants treated with β-estradiol (Fig. 7B). Upon induction of OsCV, *OsCV-GFP* plants decrease their photosynthesis and chlorophyll content (Fig. 7C and D, respectively). In contrast to RNAi*CV* plants, the *EST::CV-GFP* plants displayed a decrease in the primary N assimilation processes represented by lower *NR* and higher *GDH* gene expression and enzymatic activities (Figs 7E, F and 6G, H, respectively).

### OsCV interacts with the stromal glutamine synthetase 2 *in vivo*

**Fig. 8.**
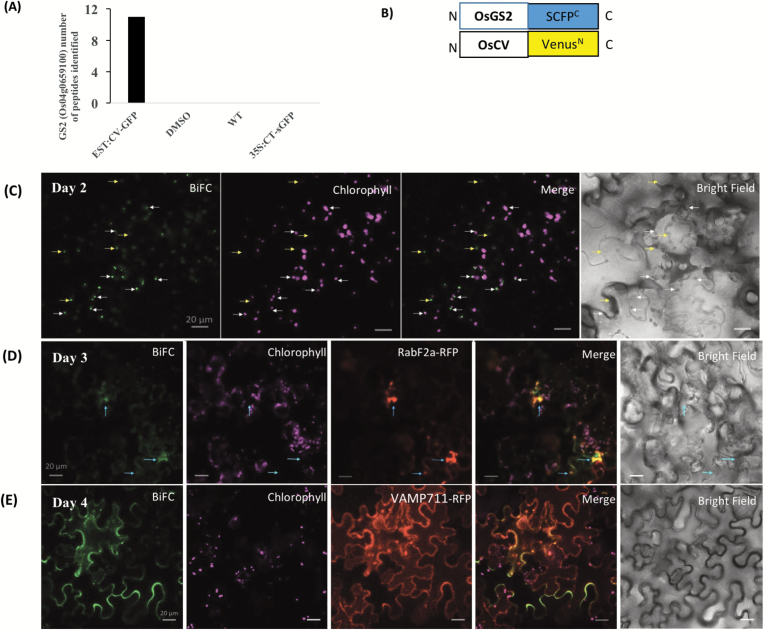
OsCV interacts with the chloroplast stromal glutamine synthetase 2 *in vivo*. (A) Glutamine synthetase 2 protein interacts with OsCV as identified by co-immunoprecipitation and MS. (B) BiFC design: SCFP^C^ and Venus^N^ are fused at the C-terminus of OsGS2 and OsCV, respectively. (C–E) BiFC analysis of *in vivo* interactions: OsCV–Venus^N^ and OsGS2–SCFP^C^ (CV+GS2), by transient expression in *Nicotiana benthamiana*. (C) BiFC signals obtained 2 d after infiltration. Most of the signals overlapped with chloroplasts. White arrows indicate BiFC signal outside the chloroplast. Yellow arrows indicate BiFC signal in chloroplasts. (D) BiFC signals obtained 3 d after infiltration. Signals overlapped with chloroplasts and the PVC marker (RabF2a–RFP). (E) BiFC signals obtained 4 d after infiltration. Signals overlapped with the vacuolar marker (VAMP711–RFP).

To elucidate the mechanism(s) by which OsCV might affect N assimilation, we identified potential CV-interacting proteins by co-immunoprecipitation and subsequent identification of proteins interacting with OsCV. Immunoprecipitated CV–GFP and its interacting proteins from total protein extracts were obtained from β-estradiol-treated transgenic plants expressing OsCV–GFP. Protein extracts from wild-type plants, *EST::CV-GFP* plants without induction (DMSO) and *35S::CT-sGFP* (stromal target GFP) were used as a control to detect proteins that bind non-specifically to the anti-GFP beads. The stromal glutamine synthetase 2 (GS2) was repeatedly detected by LC-MS/MS only in the *EST::CV-GFP* plants (Fig. 8A). BiFC was used to confirm the interaction. The transient expression of fusion genes *OsCV- Venus*^*N*^ and *GS2- SCFP*^*C*^ in *N. benthamiana* resulted in BiFC fluorescence (Fig. 8C–E), confirming that the *in vivo* interaction between OsCV and OsGS2 occurs in chloroplasts (Fig. 8C; Supplementary Fig. S2A). To assess whether the complex OsCV–OsGS2 was eventually transported to vacuoles, the BiFC constructs were transiently expressed with Rab*2a-RFP*, a pre-vacuolar compartment (PVC) *rab5 GTPase Rha1* (Foresti *et al.*, 2010), and *VAMP711-RFP* encoding a tonoplast R-SNARE (Uemura *et al.*, 2004) in *N. benthamiana* leaves. Our results showed that the CV–GS2 BiFC signal interaction overlapped with RabF2a–RFP at 3 d and VAMP711–RFP at 4 d after infiltration (Fig. 8C and D, respectively). We also transiently co-expressed *CV-CFP* together with *GS2-YFP* in *N. benthamiana* leaves. Cells that co-expressed both *CV-CFP* and *GS2-YFP* showed the co-localization of both proteins at chloroplasts (Supplementary Fig. S2A), outside of chloroplasts (Supplementary Fig. S2A), in the PVC (Supplementary Fig. S2B), and in the vacuole (Supplementary Fig. S2C). In cells expressing only GS2–YFP or CV–CFP 4 d after infiltration, GS2 was mostly localized to chloroplasts (Supplementary Fig. S3A), while CV was mostly localized outside of chloroplasts (Supplementary Fig. S3B). Collectively, these results indicate an interaction of OsCV with GS2 in chloroplasts.

## Discussion

Abiotic stress accelerates leaf senescence, chlorophyll degradation, and the loss of photosynthetic activity, and chloroplast breakdown is among the early processes that are initiated during leaf senescence (Avila-Ospina *et al.*, 2014). We have shown previously that the Arabidopsis *CV* (*AtCV*) gene encodes a protein that functions as a scaffold, targeting thylakoid-bound and stromal proteins for degradation in the plant vacuoles (Wang and Blumwald, 2014). In agreement with this, silencing of *AtCV* resulted in delayed stress-induced senescence, enhanced chloroplast stability, and increased stress tolerance in Arabidopsis (Wang and Blumwald, 2014). Interestingly, while autophagy (Ishida and Yoshimoto, 2008) mediates general cellular degradation, CV is specific for chloroplast degradation (Wang and Blumwald, 2014). Since the chloroplasts contain up to 70% of the leaf N, they are the main source for nutrient mobilization during senescence (Diaz-Mendoza *et al.*, 2016). Given the co-ordinated regulation of N and C metabolism in plants, we queried whether manipulating chloroplast stability, delaying the stress-induced chloroplast degradation, would result in improved source–sink relationships under water-deficit stress in a crop plant. This question is of particular relevance in annual grain crops such as rice, which is highly dependent on the remobilization of N and C pools from the source flag leaf to the grain. Interestingly, *CV* genes have been identified in all sequenced plant genomes, including *O. sativa* (Wang and Blumwald, 2014). We hypothesized that down-regulating the expression of the rice *CV* (*OsCV*) would delay the stress-induced chloroplast degradation, maintaining photosynthesis in the source leaves and enhancing source fitness (enhanced C and N metabolism), contributing to the production of the N and C skeleton to be allocated to the grain upon filling.

The correlation of *OsCV* expression patterns with both plant age and exposure to stress (Fig. 1) and the ubiquity of *CV* in plants (Wang and Blumwald, 2014) suggested a conserved functional role in stress-induced senescence. We designed the stress treatments allowing the plants to undergo a pre-anthesis stress and water shortage cycles until the grain-filling stage in order to extend the period of enhanced C and N metabolism of the *OsCV*-silenced plants over wild-type plants.

Whether delaying plant senescence via increasing chloroplast stability is a beneficial trait for increasing yield properties and source–sink relationships is not clear, since delayed senescence, albeit leading to increased source fitness, could also affect N and nutrient remobilization to the sink (Gregersen *et al.*, 2014; Jagadish *et al.*, 2015). Our results, showing that the down-regulation of *CV* led to better yield performance under water-deficit conditions (Fig. 2), would support the beneficial effects of increasing chloroplast stability and delayed senescence on yield productivity in grain crops under stress. This notion was also supported by our previous work showing enhanced crop water-deficit tolerance by delaying senescence through the stress-induced synthesis of CKs and the alteration in C and N homeostasis (Peleg *et al.*, 2011). In addition, the enhanced chloroplast stability and photosynthetic capacity under water-deficit stress displayed by RNAi*OsCV* indicated the role of OsCV in chloroplast degradation and stress tolerance (Fig. 3). We did not find a significant increase in the dry biomass of RNAi*OsCV* plants under our experimental conditions, although a trend was noted (Fig. 2D). Rice biomass accumulation is more pronounced during early vegetative growth (Luh, 1991); however, our stress treatments started at the mid to late vegetative growth. Thus significant differences in dry biomass (excluding panicles) should not be expected.

Whole-leaf transcriptome analysis of wild-type and RNAi*OsCV* plants revealed significant differences in the expression of genes encoding proteins associated with photosynthesis, N and amino acid metabolism, and chloroplast organization and structure under water stress (Fig. 4). These differences suggested that RNAi*OsCV* plants were able to maintain C and N assimilation during stress. RNAi*OsCV* plants under water-deficit stress displayed a lower inhibition of photosynthetic activity than wild-type plants, as shown by the expression of photosynthesis-related genes (Fig. 5A), higher CO_2_ assimilation rates (Fig. 5B), and higher amounts of metabolites originating during photosynthesis (Fig. 5C). In addition, the presence of higher levels of metabolites such as dihydroxyacetone and erythrose in RNAi*OsCV* plants would enhance the ribulose bisphosphate regeneration capacity during water-deficit stress conditions and maintain C assimilation. There are a number of N assimilation processes that take place mainly in the chloroplasts and can affect source fitness and photosynthesis (Masclaux-Daubresse *et al.*, 2010; Nunes-Nesi *et al.*, 2010).

During water-deficit stress, RNAi*OsCV* plants increased N assimilation that was mediated by higher NR activity and higher expression of genes encoding enzymes associated with the GS2/GOGAT cycle (Fig. 6A, C, E; Supplementary Fig. S1). In contrast, the wild type displayed the accumulation of free amino acids, together with higher expression of cytosolic *GS1* and *AS* and mitochondrial *GDH* (Fig. 6A, B; Supplementary Fig. S1). GS1 and AS have been reported to play important roles in the regulation of phloem amino acid remobilization from source to sink upon senescence (Masclaux-Daubresse *et al.*, 2008, 2010). *GDH* expression and GDH-mediated deamination activity, known to be induced in leaves during stress and to mediate N remobilization (Diaz *et al.*, 2008; Masclaux-Daubresse *et al.*, 2008, 2010) was also induced during *OsCV* overexpression (Fig. 7D, F). Under stress conditions, GDH could supply ammonium to the GS/GOGAT cycle and facilitate C recycling via 2-oxoglutarate (Reguera *et al.*, 2013). Because of the toxicity of free ammonium, a fast conversion of NH_4_ into amino acids is required in order to avoid deleterious effects and to provide nitrogenous forms suitable for source–sink transport. On the other hand, the accumulation of free amino acids in the wild type during stress could be the result of: (i) a proteolytic process, frequently described under stress conditions (Fig. 6B); or (ii) insufficient C to be used along with N for growth and development (Paul and Driscoll, 1997; Stitt and Krapp, 1999). Our results would indicate that under stress conditions, wild-type plants enhanced senescence and the degradation of chloroplastic proteins, leading to a premature N remobilization in the form of amino acids (Havé *et al.*, 2017), from source leaves to sinks (i.e. seeds) with a concomitant grain yield penalty.

Another pathway important for N assimilation is photorespiration. In C_3_ plants, enhancing photorespiration resulted in increased N assimilation via NR (Rachmilevitch *et al.*, 2004; Bloom, 2015). Differences in the glyoxylate metabolism pathway were seen between RNAi*OsCV* and wild-type plants (Fig. 4A). RNAi*OsCV* plants exhibited enhanced expression of genes encoding proteins associated with photorespiration and increased metabolite contents under water-deficit stress (Supplementary Fig. S3; Supplementary Table S4). It is plausible that under stress, chloroplast stability led to increasing photorespiration and the production of reducing power required for NR activity (Rachmilevitch *et al.*, 2004). Our observations are in agreement with a previous report showing that tobacco plants overproducing CKs displayed chloroplast stability leading to enhanced photorespiration and increased stress tolerance (Rivero *et al.*, 2009). Interestingly, the constitutive *OsCV* silencing led to increased *OsNR* expression and higher OsNR activity under well-watered conditions (Fig. 6B). Rice overexpressing isopentenyltransferase (IPT; with the resulting increase in CKs) also displayed enhanced NR activity under well-watered conditions (Reguera *et al.*, 2013). These results would suggest possible roles for OsCV in the regulation of N assimilation. Future experiments where *OsCV* silencing is driven by a stress-inducible promoter could shed light on the different roles of OsCV. In contrast to the increased N and C assimilation displayed by RNAi*OsCV* plants under stress, the overexpression of *OsCV* (Fig. 7A, B) resulted in a significant decrease in photosynthesis (Fig. 7C), chlorophyll content (Fig. 7D), and N assimilation (Fig. 7E, G) that was accompanied by increased GDH activity and *GDH* expression (Fig. 7F, H).

Our results support the notion of OsCV involvement in N assimilation. The manipulation of *OsCV* expression affected primary N assimilation and cytosolic NR, although a direct relationship between chloroplast stability and NR activity is not yet clear. The interaction between OsCV and chloroplastic N assimilation enzymes is a possible mechanism by which OsCV regulates N assimilation. Co-immunoprecipitation and subsequent analysis by LC-MS/MS revealed the possible interaction between OsCV and GS2 (Fig. 8A). The interaction was confirmed *in vivo* by co-localization and BiFC assays (Fig. 8; Supplementary Fig. S2). Moreover, the OsCV–GS2 interaction and co-localization was also observed at the PVC and vacuole (Fig. 8; Supplementary Fig. S2). These results indicated that CCVs contained OsGS2 and were transported together to the vacuole for recycling. GS2 is a major enzyme for N assimilation, and *GS2* overexpression leads to increased stress tolerance in rice (Hoshida *et al.*, 2000). Moreover, an increase in GS2 was correlated with increased protection of the photosynthesis apparatus via the enhancement of photorespiration (Hoshida *et al.*, 2000), a phenomenon which was also observed in the RNAi*OsCV* plants (Supplementary Fig. S4, Table S4). This notion was also supported by the increased expression of GS2 (Supplementary Fig. S1C), which may imply a higher activity of GS2 leading to a higher N assimilation in chloroplasts, photorespiration, and eventually stress tolerance. It is possible that in addition to thylakoid-associated proteins (Wang and Blumwald, 2014), OsCV targeted stromal proteins associated with N metabolism for further degradation through the CV-mediated pathway. Although a significant amount of research addressed the pathways of Rubisco senescence-associated degradation (Hortensteiner and Feller, 2002; Prins *et al.*, 2008), the pathway(s) by which abundant chloroplastic proteins such as GS2 and Fd-GOGAT are targeted remain to be elucidated (Thoenen and Feller, 1998; Hortensteiner and Feller, 2002). Although the role of SAVs and Rubisco-containing bodies (RCBs) in GS2 degradation was suggested, since GS2 protein was enriched in isolated SAVs (Martinez *et al.*, 2008) and in RCBs (Chiba *et al.*, 2003), our results would indicate that the OsCV-mediated chloroplast degradation pathway is directly involved in regulating N assimilation during stress-induced senescence.

## Supplementary data

Supplementary data are available at *JXB* online.

Table S1. List of primers used in qPCR.

Table S2. Full gene names, *P*-values, and expression values for photosynthesis-associated genes.

Table S3. Full gene names, *P*-values, and expression values for nitrogen metabolism-associated genes.

Table S4. Full gene names, *P*-values, and expression values for photorespiration-associated genes.

Fig. S1. Glutamine synthetase activity and expression

Fig. S2. Confocal microscopy observations of *Nicotiana benthamiana* cells transiently co-expressing CV–CFP, GS2–YFP, and PVC and vacuolar markers.

Fig. S3. Confocal microscopy observations of *Nicotiana benthamiana* cells transiently expressing CV–CFP and GS2–YFP.

Fig. S4. Down-regulation of OsCV resulted in enhanced photorespiration under water stress.

## Supplementary Material

Supplementary Tables S1-S4_Figures S1-S4

## Abbreviations:

ABA: abscisic acid
AS: aspargine synthetase
CK: cytokinin
CV: CHLOROPLAST VESICULATION
GDH: glutamate dehydrogenase
GS: glutamine synthetase
NR: nitrate reductase
SARK: senescence-associated receptor kinase.
