# Supplementary material for: Delaying chloroplast turnover increases water-deficit stress tolerance through the enhancement of nitrogen assimilation in rice
Source: J Exp Bot. 2017 Jul 27;69(4):867–78. doi: 10.1093/jxb/erx247 (PMC5853860; doi:10.1093/jxb/erx247)
Supplement: Supplementary Tables S1-S4_Figures S1-S4 [file erx247_suppl_supplementary_tables_s1-s4_figures_s1-s4.docx]

**Supplementary Tables S1.** List of primers used in qPCR.

| **GENE NAME** | **ACCESION NUMBER** | **PRIMER** | **PRIMER SEQUENCE** |
| --- | --- | --- | --- |
| e-Green fluorescence protein (eGFP) | KY129798.1 | Forward | CACATGAAGCAGCACGACTT |
|  |  | Reverse | GGTCTTGTAGTTGCCGTCGT |
| Chloroplast vesiculation (CV) | Os05g0575000 | Forward | GGCTGCTTCTCCCTCTAAACG |
|  |  | Reverse | CAAATGCCATGCTCGATCGTG |
| Nitrate Reductase (NR) | Os08g0468100 | Forward | CGAGAAGCTCATCTGGAATCTCA |
|  |  | Reverse | AGCCCGATCTCACCCTTGT |
| Glutamate dehydrogenase (GDH) | Os04g0543900 | Forward | GTTCTCATCCCATGCGCTTTAG |
|  |  | Reverse | GCCTTCACATCAGGTGCATTT |
| Cytoplasmic glutamine synthetase (GS1) | Os03g0712800 | Forward | CCCCTTCACCGACAAGATCA |
|  |  | Reverse | AGGTCAATTCCAGTTCCTCCAA |
| Chloroplastic glutamine synthetase (GS2) | Os04g0659100 | Forward | CGAGAAGGACGGCAAAGGTTAC |
|  |  | Reverse | TCCTCACTTGCCGTTGGATTGG |
| Transcription elongation factor (TEF) | Os03g0177400 | Forward | GACCCGTGAGCACGCTCTT |
|  |  | Reverse | TGGCATCCATCTTGTTGCA |

**Supplementary Tables S2**. Full genes names, p values and expression values for photosynthesis related genes

| ID | Gene name | wt drought VS  CV drought fold change | adjusted p value |
| --- | --- | --- | --- |
| os09g0411650 | Ribulose-1,5 bisphosphate carboxylase oxygenase large subunit N-methyltransferase | -1.2672099 | 0.00181805 |
| os12g0274700 | RIBULOSE BISPHOSPHATE CARBOXYLASE SMALL CHAIN 1A | -1.7904245 | 0.001705374 |
| os04g0459500 | GLYCERALDEHYDE 3-PHOSPHATE DEHYDROGENASE A SUBUNIT | -1.4296721 | 0.000989884 |
| os03g0129300 | GLYCERALDEHYDE-3-PHOSPHATE DEHYDROGENASE B SUBUNIT | -1.3557587 | 0.000600005 |
| os03g0267300 | Fructose-1,6-bisphosphatase | -1.3077013 | 0.00175143 |
| os04g0234600 | Sedoheptulose-bisphosphatase | -0.86175346 | 0.001262001 |
| os01g0869800 | NONPHOTOCHEMICAL QUENCHING 4 | -1.3820374 | 0.000600005 |
| os01g0617900 | PsbP-related thylakoid lumenal protein 4 | -0.39697224 | 0.00247028 |
| os07g0141400 | PHOTOSYSTEM II SUBUNIT P-1 | -0.78871405 | 0.041380926 |
| os01g0501800 | PHOTOSYSTEM II SUBUNIT O-2 | -0.7539296 | 0.002048087 |
| os08g0347500 | PsbP-like protein 1 | -0.79665077 | 0.002163034 |
| os01g0720500 | LIGHT-HARVESTING CHLOROPHYLL-PROTEIN COMPLEX II SUBUNIT B1 | -2.353972 | 0.014815246 |
| os11g0242800 | LIGHT HARVESTING COMPLEX OF PHOTOSYSTEM II 5 | -1.1432285 | 0.001651588 |
| os02g0764500 | PHOTOSYSTEM I LIGHT HARVESTING COMPLEX GENE 5 | -1.2838906 | 0.000619963 |
| os04g0457000 | LIGHT HARVESTING COMPLEX PHOTOSYSTEM II SUBUNIT 6 | -1.7834424 | 0.003214614 |
| os07g0558400 | LIGHT HARVESTING COMPLEX PHOTOSYSTEM II | -1.5556113 | 0.000643986 |
| os07g0562700 | LIGHT-HARVESTING CHLOROPHYLL B-BINDING PROTEIN 3 | -0.94257176 | 0.00143561 |
| os03g0778100 | Photosystem I subunit F | -0.90479714 | 0.019926186 |
| os12g0420400 | Photosystem I subunit L | -0.7410059 | 0.004713307 |
| os08g0560900 | Photosystem I subunit D-1 | -0.80083686 | 0.002707027 |

**Supplementary Tables S3**. Full genes names, p values and expression values for nitrogen metabolism related genes

| ID | Gene name | wt drought VS CV drought fold change | adjusted p value |
| --- | --- | --- | --- |
| os01g0547600 | Nitrate transporter2 | -2.326907 | 0.04035707 |
| os08g0468100 | NITRATE REDUCTASE 1 | -1.7153509 | 0.002236371 |
| os04g0659100 | GLUTAMINE SYNTHETASE 2 | -1.559191 | 0.001254879 |
| os03g0712800 | GLUTAMINE SYNTHETASE 1 | 2.0769646 | 0.002238914 |
| os07g0658400 | Glutamate synthase (ferredoxin) | -1.1520258 | 0.002132439 |
| os04g0543900 | GLUTAMATE DEHYDROGENASE 2 | 0.745512 | 0.005781014 |
| OS01G0357100 | Nitrite reductase (ferredoxin) | -0.805507217 | 0.108987504 |
| OS03G0291500 | Asparagine synthetase | 3.063022997 | 0.053051551 |

**Supplementary Tables S4**. Full genes names, p values and expression values for photorespiration related genes

| ID | Gene name | wt drought VS CV drought fold change | adjusted p value |
| --- | --- | --- | --- |
| os06g0611900 | glycine decarboxylase P-protein 2 | -1.2856581 | 0.000644 |
| os10g0516100 | glycine cleavage system H protein | -2.2196546 | 0.001736 |
| os03g0738400 | SERINE TRANSHYDROXYMETHYLTRANSFERASE 1 | -1.1958693 | 0.000745 |
| os08g0502700 | ALANINE:GLYOXYLATE AMINOTRANSFERASE | -0.70863557 | 0.004306 |
| os07g0152900 | GLYCOLATE OXIDASE 1 | -1.0568849 | 0.000633 |
| os07g0616500 | (S)-2-hydroxy-acid oxidase | -0.6080312 | 0.007879 |
| os02g0101500 | NADH-dependent hydroxypyruvate reductase | -0.6027539 | 0.001205 |
| os12g0291400 | RIBULOSE BISPHOSPHATE CARBOXYLASE SMALL CHAIN 1A | -1.3179345 | 0.004335 |


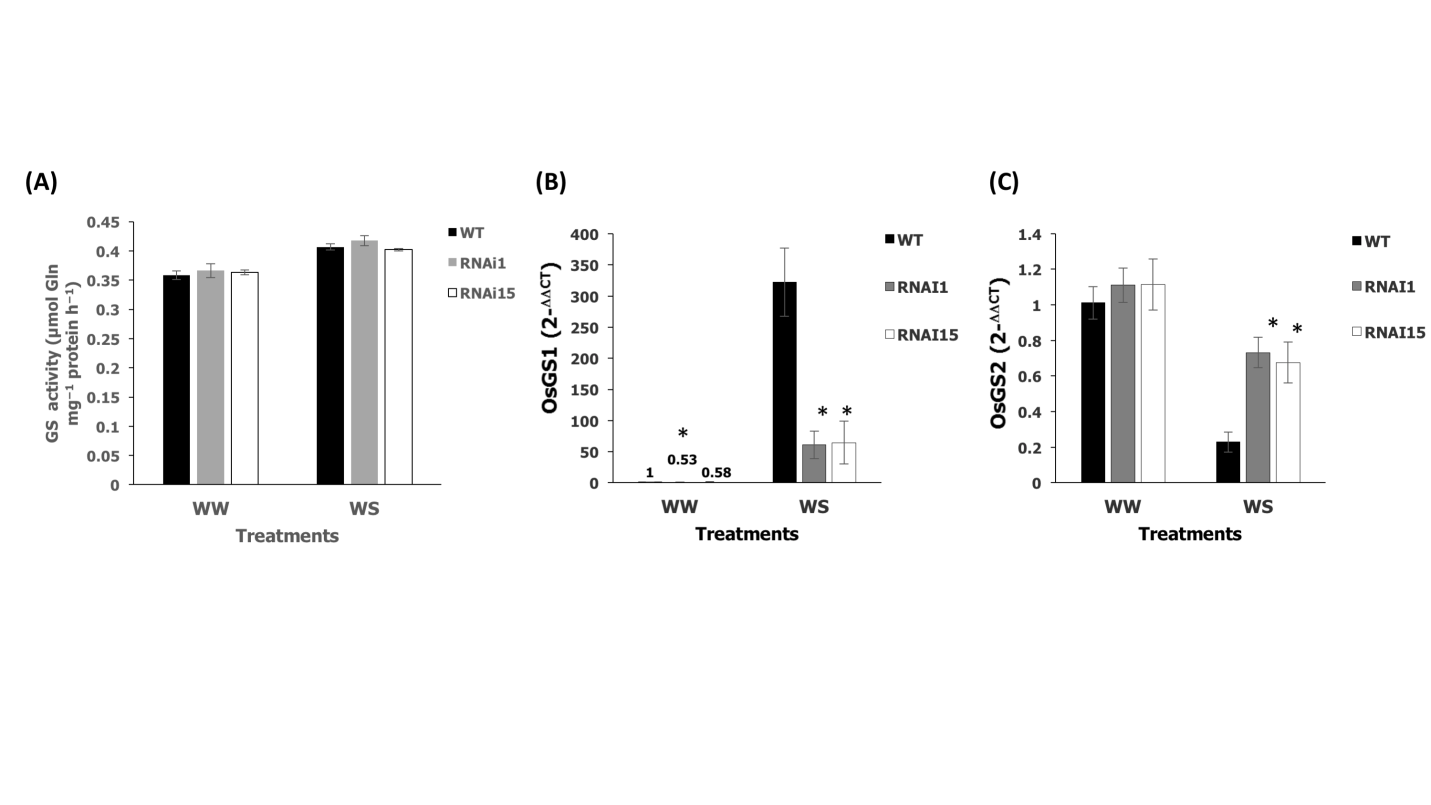


Supplemental Fig. 1. GLUTAMINE SYNTHETASE activity and expression

Analysis of (A) activity (B) expression of GLUTAMINE SYNTHETASE 1 and (C) expression of GLUTAMINE SYNTHETASE 2 in WT and *RNAiOsCV* plants (lines RNAi1 and RNAi15). Values are Mean ± SE (n = 4 biological repetitions). The data were analyzed using Student’s t test. Asterisks indicate significant differences from WT for each treatment (P ≤ 0.05).


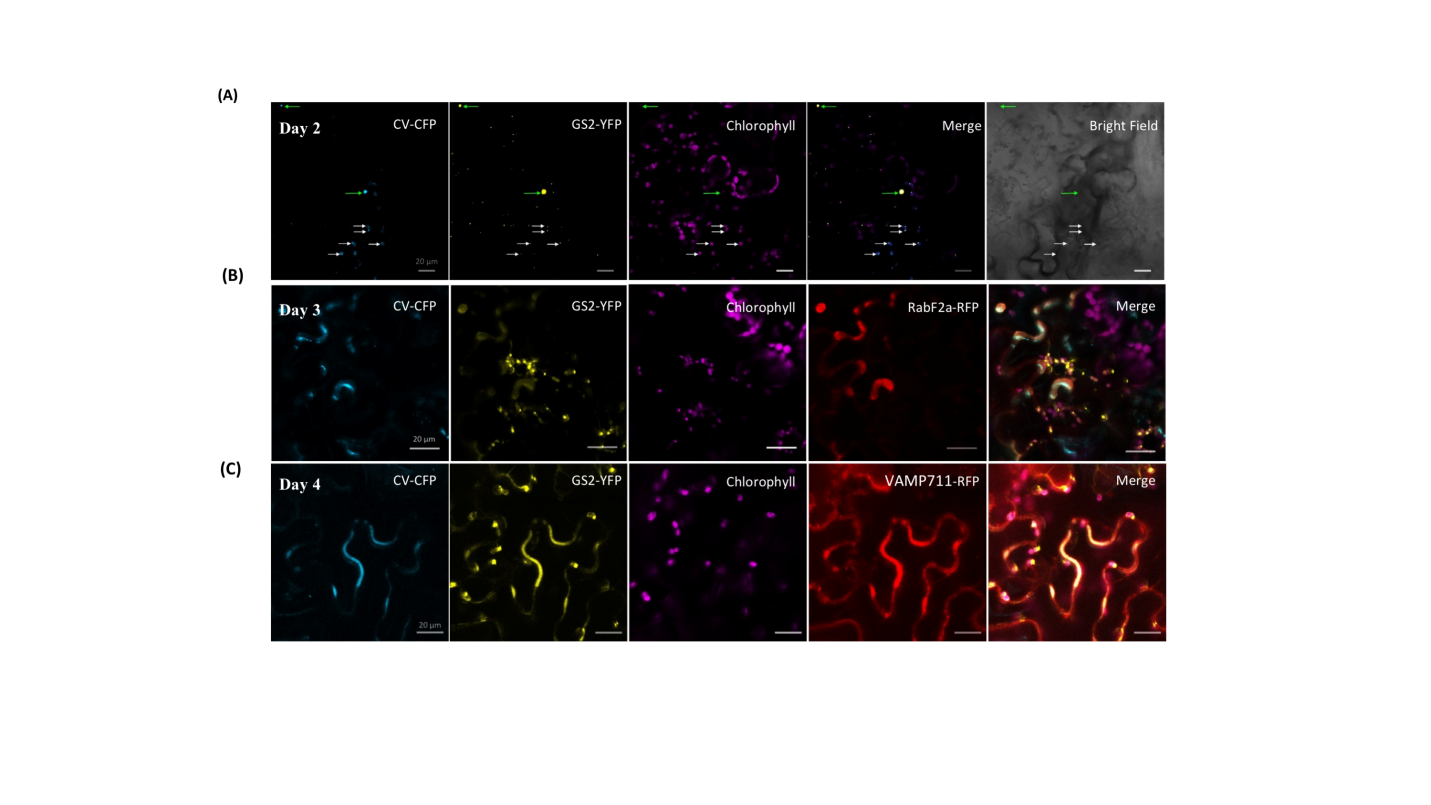


**Supplementary Figure S2.** Confocal microscopy observations of of *Nicotiana benthamiana* cells from transiently co-expressing CV-CFP, GS2-YFP, PVC marker and vacuolar marker

(A) Signals 2 days after infiltration. Most of the signals are overlapped with chloroplasts. Green arrows indicate CV-YFP and GS2-CFP co- localized outside the chloroplast. White arrows indicate CV-YFP and GS2-CFP co- localized in chloroplast

(B) Signals 3 days after infiltration. Signals are overlapped with chloroplasts and PVC marker (RabF2a-RFP).

(C) Signals 4 days after infiltration. Signals are also overlapped with vacuolar marker (VAMP711-RFP).


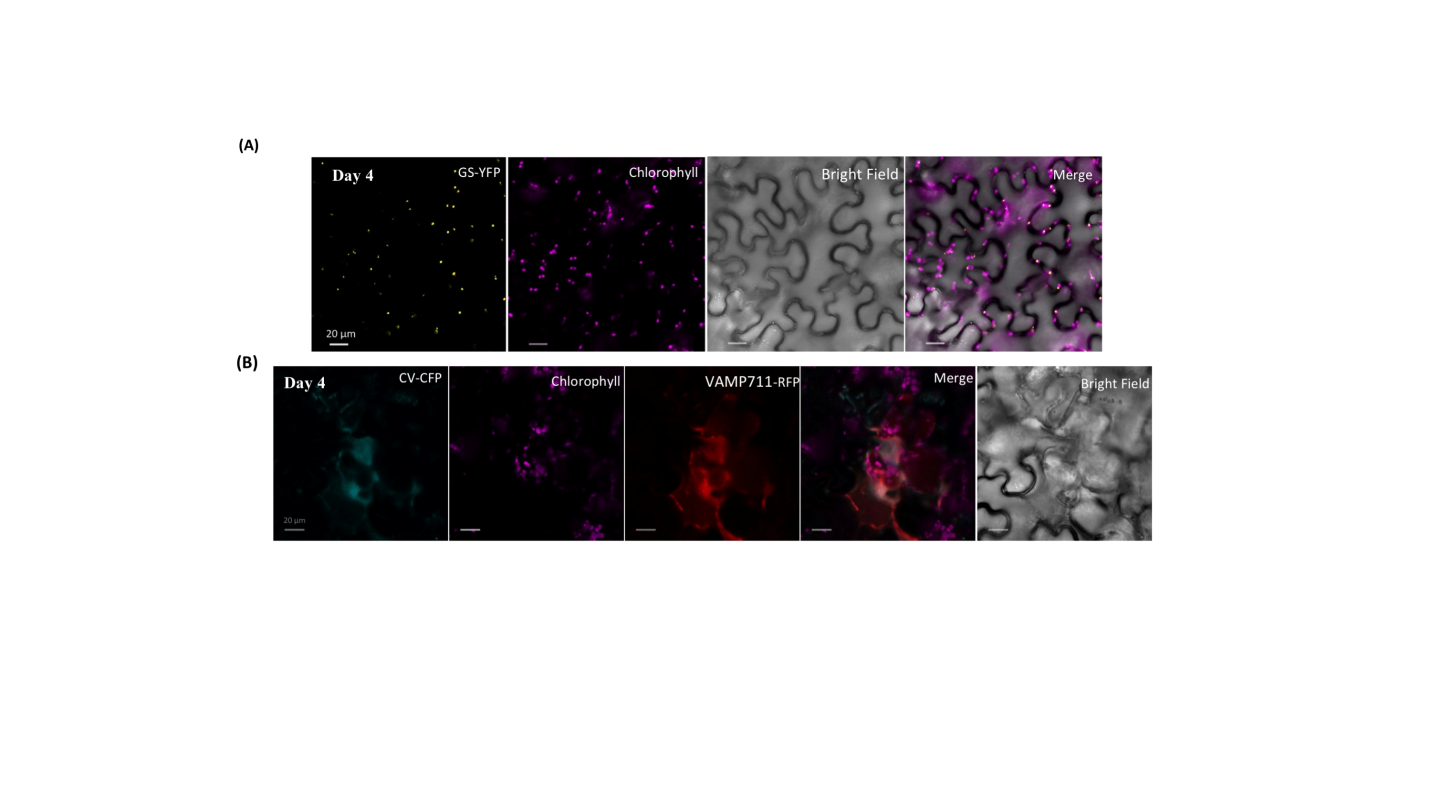


**Supplementary Figure S3.** Confocal microscopy observations of of *Nicotiana benthamiana* cells from transiently expressing CV-CFP, GS2-YFP.
(A) Signals of GS2-YFP 4 days after infiltration. Signals are overlapped with chloroplasts.

(B) Signals of CV-CFP 4 days after infiltration. Signals are overlapped with vacuole.


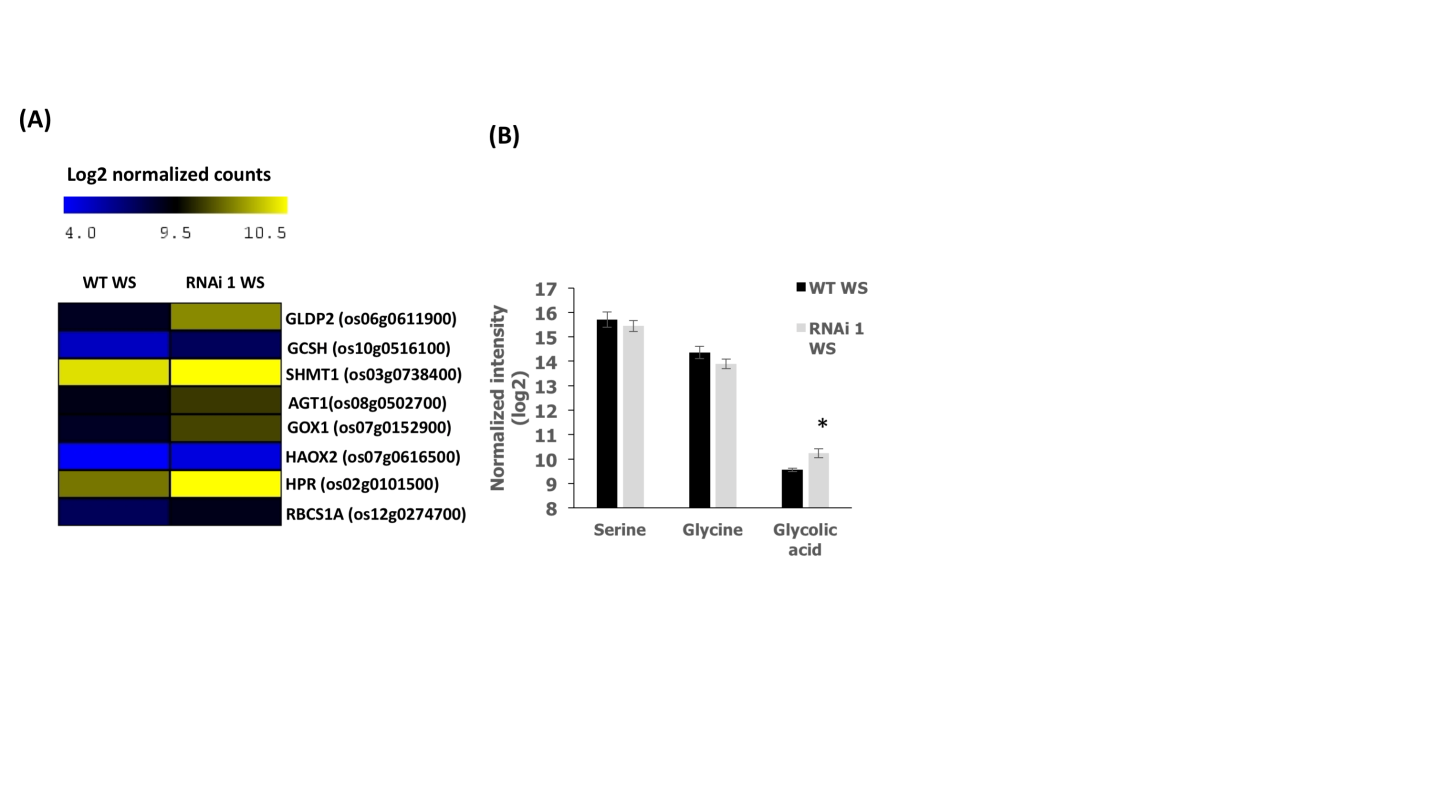


**Supplementary Figure S4.** Downregulation of OsCV resulted in enhanced photorespiration under water stress

(A) Heatmap representation of significant selected main photorespiration related genes (for full genes names, p values and expression values see Supplementary Tables S4) in WT and RNAi plants under water stress.

(B) Selected main photorespiratory metabolites of leaf tissue in WT and RNAi plants under water stress

The data were analyzed using Student’s t test. Asterisks indicate significant differences from WT for each treatment (P ≤ 0.05).

Error bars show SE ( n= 3-4 biological repetitions)
